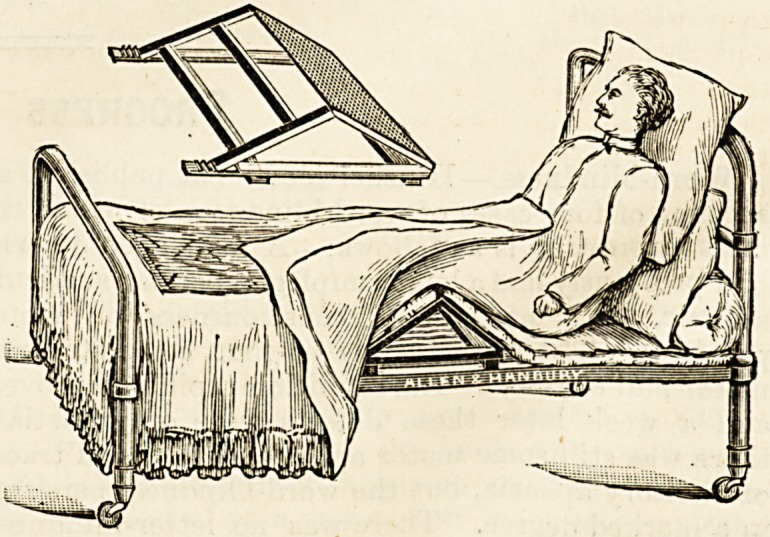# New Appliances and Things Medical

**Published:** 1902-09-06

**Authors:** 


### NEW APPLIANCES AND THINGS MEDICAL.

**[We shall be glad to receive at our Office, 28 & 29 Southampton Street, Strand, London, W.C., from the manufacturers, specimens of all new preparations and appliances which may be brought out from time to time.]**

### "SISTER DORIS" BED-REST.

(Allen & Hanburys, Limited, 48 Wigmore Street, London, W.)

The bed-rest of which we here give an illustration is a very effective contrivance, the object of which is to enable a patient to be comfortably propped up in bed. Of course it is easy enough to prop a patient up if one has at hand a supply of good firm pillows, the difficulty is to keep him propped. The slightest consideration of the mechanical principles involved is sufficient to show that a body reared up against such an inclined plane as is presented by any ordinary bed-rest or any possible combination of pillows, is sure to slip down, unless some point of counter-pressure be provided, a fact with which every nurse, without any consideration of mechanical principles at all, is sadly familiar, and the object which the "Sister Doris" bed-rest thoroughly fulfils is to provide the necessary *point d'appui*. The accompanying illustration explains the mechanism, which is simplicity itself. It is intended that the "rest" should be placed beneath the mattress, and when thus placed it is evidently capable of converting any single bed, or, indeed, any bed which is provided with a narrow mattress, into a so-called Bombay lounge. The construction of the apparatus is exceedingly simple—in fact, so simple that we can hardly persuade ourselves that it has yet attained its fullest possible development—and this simplicity has this advantage that, after the bed-rest has been used a thorough scrub and a coat of enamel paint will make it "equal to new" again. It certainly is a most effectual contrivance.

## Figures and Tables

**Figure f1:**